# Brain‐age prediction in autism: Translating imaging biomarkers without overstating clinical meaning

**DOI:** 10.1002/ped4.70077

**Published:** 2026-07-25

**Authors:** Weerapong Lilitwat

**Affiliations:** ^1^ Department of Pediatrics Texas Tech University Health Sciences Center Lubbock Texas USA

To the Editor:

Hu et al.^1^ make a meaningful contribution by demonstrating that routine T1‐ and T2‐weighted magnetic resonance imaging (MRI) can predict brain age in children aged 2–6 years and detect maturational differences in autism spectrum disorder (ASD).^1^ Using clinically available sequences rather than research‐grade protocols is a key strength, bringing this approach closer to real‐world pediatric practice. As these models gain traction, an equally urgent question emerges: how should brain age findings be communicated to clinicians and families?

The term “brain age” is intuitive but interpretively hazardous. Parents may read a delayed estimate as a diagnostic label, a proxy for intelligence, or a fixed prognosis—none of which it represents. In ASD, where developmental trajectories are heterogeneous and phenotypic diversity is the norm,^2^, ^3^ deterministic framing of a group‐derived statistic risks increasing parental anxiety and undermining engagement with early intervention—the services with the strongest evidence for improving outcomes.^2^

Several features of the study support interpretive caution. The age‐matched analysis showed significantly lower brain age difference (BAD) in ASD (mean −0.224 *vs*. 0.011 years; *P* < 0.001), yet age‐stratified differences did not survive correction for multiple comparisons, and wide BAD distributions in both groups indicate substantial individual‐level overlap.^1^ A statistically significant group effect that is individually indeterminate is precisely the finding requiring the most careful language in clinical communication. The cohort was also not stratified by symptom severity, adaptive functioning, language level, or comorbidities—dimensions that profoundly shape brain maturation and treatment trajectory in ASD^3^, ^4^—further constraining individual‐level applicability.

Future studies should integrate family‐centered reporting standards from the outset: presenting findings as probabilistic and group‐derived, reporting the full BAD distribution to convey individual overlap, and positioning brain age explicitly as complementary to developmental history, standardized behavioral assessment, and longitudinal follow‐up—not a replacement for any of them.^2^ Language implying that a single MRI‐derived number determines a child's developmental future must be avoided.^5^ Prospective studies should also assess how clinicians communicate these estimates and how families interpret them.

Hu et al.^1^ have shown that routine MRI can illuminate the neurodevelopmental landscape of early childhood ASD. The next steps—multicenter replication, longitudinal tracking, and ASD subtype analysis—are technical. But the equally essential work is communicative: building the language and standards by which these findings reach families without overstating what a group‐level biomarker can say about any one child. A biomarker earns clinical value only when it improves care without overstating certainty.

## CONFLICT OF INTEREST

The author declares no conflict of interest.
